# Managing technological sovereignty: a systematic review of semiconductor industry policy and regional ecosystem governance

**DOI:** 10.3389/frma.2026.1762083

**Published:** 2026-03-06

**Authors:** Jingwen Cai, Xuexian Fang, Yifen Yin, Yuanyuan Yu, Chunning Wang, Wai In Ho, Haoqian Hu

**Affiliations:** 1 Faculty of Humanities and Social Sciences, Macao Polytechnic University, Macao, Macao SAR, China; 2 School and Hospital of Stomatology, Guangdong Engineering Research Center of Oral Restoration and Reconstruction and Guangzhou Key Laboratory of Basic and Applied Research of Oral Regenerative Medicine, Guangzhou Medical University, Guangzhou, China

**Keywords:** geopolitics, innovation ecosystem governance, multi-level fit, semiconductor industry, supply chain resilience, technological sovereignty

## Abstract

**Introduction:**

As the semiconductor industry shifts from a logic of efficiency to one of technological sovereignty and supply chain resilience, engineering managers and policymakers face unprecedented uncertainty. While nations are relaunching industrial policies to mitigate geopolitical risks, a critical puzzle remains: why do similar macro-strategies yield divergent outcomes across different regional innovation ecosystems? Existing literature tends to bifurcate between macro-level state competition and micro-level firm strategies, creating a theoretical disconnect.

**Methods:**

Drawing on Merton's Middle-Range Theory, this study bridges this gap by adopting a “structure-process-function” perspective. We conducted a systematic review of 104 core articles from the Web of Science Core Collection to diagnose the meso-level governance mechanisms that mediate national strategy and regional context. We propose a “dual fit” analytical framework, arguing that policy effectiveness is contingent upon two simultaneous alignments: (1) “strategy-execution fit” (macro-meso), where governance mechanisms (process) must align with national security goals (structure); and (2) “execution-context fit” (meso-micro), where interventions must be embedded within the region's specific endowments and dynamic capabilities.

**Results:**

Our findings identify two primary failure modes: “governance failure” (misalignment of incentives) and “contextual failure” (neglect of absorptive capacity).

**Discussion:**

This study contributes to engineering management theory by providing a multi-level mechanism to diagnose policy-ecosystem fit, offering actionable insights for managing semiconductor supply chains in a fragmented global order.

## 1 Introduction

The semiconductor industry is rapidly becoming the core arena for global technological competition and geopolitical rivalry. As the cornerstone of the modern digital economy, the strategic value of semiconductor integrated circuits has transcended its economic attributes, evolving into a key indicator of national security and technological sovereignty (Svaton, 2025; Edler et al., 2023). For a long time, the industry relied on a highly globalized, finely specialized supply chain system (Gupta and Sharma, 2024); however, its vulnerability has been starkly exposed in recent years by the rise of techno-nationalism and persistent supply chain disruptions (Aoyama et al., 2024). In response, the world's major economies are shifting from a “market-first” to a “security-first” policy paradigm (Bown and Wang, 2024), unleashing a new wave of state intervention characterized by massive financial incentives and mandatory tools (Donnelly, 2023). For example, the CHIPS and Science Act enacted by the United States aims to drive the “reshoring” of advanced manufacturing through substantial subsidies and tax credits (Gupta and Sharma, 2024; Stokols and Kollar, 2025). The European Union (EU), similarly, is pursuing “technological sovereignty,” seeking to bolster its position in the global supply chain (Dieter and Biedermann, 2023; Konstari and Valkokari, 2025). Concurrently, traditional semiconductor powerhouses like China, Japan, and South Korea are continuously ramping up national-level support to secure their competitive edge in next-generation technologies (Aoyama et al., 2024; Yoon, 2023; Manning and Richter, 2023). This policy shift toward “autonomy” or “autarky” (Dieter and Biedermann, 2023) signifies a national reassessment of the risks of globalized specialization. Crucially, this shift creates a new operating environment where supply chains must be “stress-tested” not only against economic fluctuations but against systemic geopolitical shocks (Ivanov, 2025).

Yet, a critical question arises: Why do similar macro-policy intentions often yield vastly different implementation effects and industrial resilience outcomes across different countries, and even across different regions within the same country? In response to this question, the existing academic literature, while offering rich insights, often presents a “polarized” analytical landscape. It tends to focus on either macro-level state competition or micro-level corporate strategies, overlooking the critical linkage that connects the two. On the one hand, a large body of research concentrates on the macro-level of international relations and geopolitics. These works incisively analyze interstate technological competition, trade conflicts, and security drivers, such as the underlying logic of the Sino-US tech war (Malkin and He, 2024) or the strategic considerations of the US-Japan technology alliance (Yoon, 2023; del Aguila, 2024). This stream of research clearly explains “why” national-level policy interventions occur, viewing them as necessary instruments for great-power competition and safeguarding national security (Svaton, 2025). However, they often stop at the declaration of macro-intentions, seldom delving into the complexities of “how” these national strategies are implemented, particularly the mechanisms of their spatial transmission.

On the other hand, another extensive body of literature focuses on micro-level firm behavior and supply chain management. These studies detail firms' strategic decisions under uncertainty (Germann et al., 2024), the dynamics of R&D investment, or the role of specific digital tools in enhancing supply chain resilience (Lin et al., 2025). Recent engineering management scholarship has further expanded this micro-perspective by modeling resilience mitigation strategies (Moktadir and Ren, 2024) or optimizing supplier selection under geopolitical risks (Lin et al., 2025). Consequently, a significant “meso-level theoretical gap” exists between macro-level national strategy and micro-level corporate response. Scholars in public administration argue that grand theories often fail to explain specific administrative outcomes due to a lack of operative mechanisms. To address this, we adopt a Mertonian Middle-Range Theory approach (Abner et al., 2017), which emphasizes the identification of intermediate causal mechanisms over abstract generalizations. Specifically, we utilize a modified “structure-process-function” (SPF) perspective. While traditional structural functionalism focuses on static equilibrium, the SPF framework—informed by process philosophy—posits that the “structure” (macro-strategy and micro-endowments) can only generate the desired “function” (resilience) through a dynamic “process” of governance (Liu and Fan, 2017). The current literature's failure to explain heterogeneous policy outcomes stems precisely from neglecting this meso-level governance process.

To systematically investigate this “meso-level gap” and construct a middle-range theoretical framework, this paper adopts the “systematic review” research paradigm. We followed the PRISMA 2020 (Preferred Reporting Items for Systematic Reviews and Meta-Analyses) process (Page et al., 2021), systematically retrieving, screening, and evaluating literature strictly from the Web of Science (WoS) Core Collection to ensure the quality and peer-reviewed nature of the evidence. A final analytical sample of 104 core articles was incorporated. Subsequently, this study employs the narrative synthesis method (Popay et al., 2006; Chiu et al., 2017) to synthesize and distill this highly heterogeneous yet substantively related body of evidence.

This study aims to answer the following set of core research questions (RQs) through a systematic synthesis of the extant evidence: (RQ1) How does the literature define the strategic role of the state (structure)? (RQ2) How does the literature analyze the policy instruments and governance mechanisms (process)? (RQ3) How does the literature assess the endowments of the regional innovation ecosystems (RIS) (contextual structure)? and (RQ4) How do the interactions among these dimensions influence policy effectiveness (function)? By answering these questions, the central argument of this review is that the effectiveness of regional semiconductor policy is a problem of “multi-level fit.” The success or failure of policy hinges upon whether “Strategy-Execution Fit” (macro-meso fit) and “Execution-Context Fit” (meso-micro fit) can be simultaneously achieved. In other words, the national strategy (macro) must be aligned with its chosen policy instruments and governance mechanisms (meso); in turn, these meso-level interventions must be embedded within the extant endowments and dynamic capabilities of the regional innovation ecosystem (micro). By constructing a “dual fit” analytical framework that integrates these three levels, this paper offers a middle-range theoretical perspective to explain the heterogeneous outcomes of current global semiconductor policy interventions.

## 2 Research design and methods

### 2.1 Research paradigm

To systematically investigate the “meso-level theoretical gap” discussed in the introduction, this study employs the systematic review research paradigm. Given that the literature on semiconductor industry policy is characterized by its highly interdisciplinary, heterogeneous (comprising theoretical, empirical, and policy reports), and time-sensitive nature, a systematic review is the optimal path for mapping the knowledge domain, identifying core variables, and comprehensively synthesizing theoretical models. This approach aligns with recent engineering management scholarship that utilizes systematic reviews to unravel complex governance mechanisms in emerging technology sectors (Nan et al., 2025). This study's methodological framework adheres to rigorous standards. First, we adopted a convergent parallel design (Creswell and Creswell, 2018) to integrate qualitative case evidence with systematic policy analysis. Second, regarding the review protocol, although not formally registered, a protocol was developed a priori to guide the search and synthesis process. This study strictly follows the PRISMA 2020 (Preferred Reporting Items for Systematic Reviews and Meta-Analyses) statement to ensure transparency and reproducibility (Page et al., 2021). Finally, we employed the narrative synthesis method to structurally deconstruct the heterogeneous body of evidence (Popay et al., 2006; Chiu et al., 2017).

### 2.2 Literature search and screening

The literature search was conducted exclusively within the WoS Core Collection, spanning the period from January 1990 to October 26, 2025, and limited to English-language publications. We selected WoS as the sole data source due to its high indexing standards for peer-reviewed journals in engineering management and political economy, ensuring the quality control of the evidence base. To ensure both comprehensiveness (recall) and relevance (precision), we designed a complex, multi-stage search strategy comprising one “core search” and three “supplementary searches.” Specific search strings included terms related to “semiconductor,” “industrial policy,” and “regional innovation” (see Table 1 for full Boolean queries). This strategy was designed to systematically capture literature related to the three levels of the semiconductor industry: macro (national strategy), meso (policy governance), and micro (regional innovation ecosystem). The detailed search strategies, logic, and results are presented in Table 1. The detailed Boolean search strings for all queries, including the specific keyword combinations for the five sub-themes in Search 3, are provided in Appendix A.

**Table 1:** Literature search strategy, logic, and screening results.

**Search batch**	**Objective and logic**	**Core concept group composition**	**Unique WoS records**	**Final number included**
Search 1	Core search: to precisely identify literature discussing the industry, meso-level policy, and regions simultaneously.	(Industry AND policy AND region)	874	64
Search 2	Supplementary search (macro-level): to capture literature using “geopolitics” or “national strategy” (macro terms) as substitutes for “policy” (meso term).	(Industry AND national strategy AND region)	17	0
Search 3	Supplementary search (non-regional perspective): to capture literature discussing policy at the national level (including macro and meso), but not explicitly mentioning “region” in the abstract.	[Industry AND (policy OR national strategy)]	3,389	33
Search 4	Supplementary search (theoretical perspective): to capture literature discussing “regional ecosystem mechanisms” (micro) from an “innovation systems” or “economic geography” theoretical standpoint.	(Industry AND innovation system mechanisms)	290	7
Total			4,570	104

Search 3 consisted of 5 sub-queries. The sum of unique, de-duplicated records was 767 + 841 + 836 + 796 + 149 = 3,389; the sum of included articles was 20 + 4 + 6 + 2 + 1 = 33.

The screening process was conducted in two stages. First, titles and abstracts were screened. Second, full texts were assessed for eligibility. To ensure reliability, full-text screening was performed independently by two researchers. Discrepancies were resolved through discussion and adjudication by a third senior researcher. Prior to formal data extraction, a pilot coding test was conducted on a random sample of 10 articles to calibrate the understanding of the macro/meso/micro categories among the research team. We strictly defined the “Unit of Analysis” (Yin, 2018) to ensure comparability: (1) the subject matter must focus on the semiconductor industry's governance or innovation activities; and (2) the analysis must explicitly involve policy interventions or regional ecosystem dynamics. The inclusion criteria were: (1) the subject matter must be directly related to the semiconductor industry's economic, policy, governance, or innovation activities; and (2) the analytic corpus was strictly limited to peer-reviewed academic journal articles and indexed conference proceedings to ensure methodological rigor. Exclusion criteria specifically included: (1) purely technical engineering papers; (2) macroeconomic reports without industrial specificity; and (3) gray literature, including institutional reports (e.g., from SIA or BCG) and non-peer-reviewed white papers. Although such reports were consulted to establish the contextual background in the Introduction, they were excluded from the systematic review sample (*N* = 104) to maintain the consistency of the evidence base.

**Figure 1:**
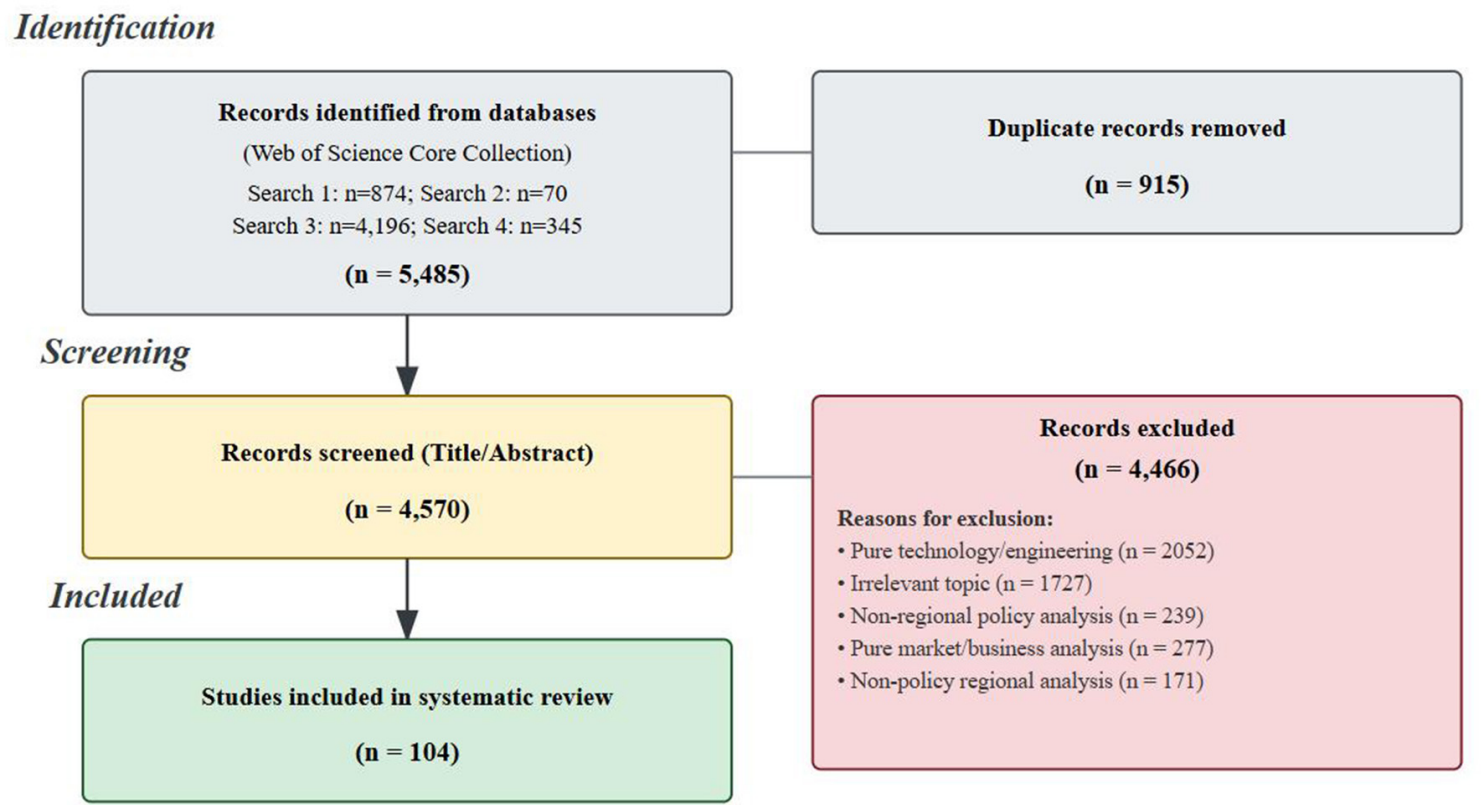
PRISMA 2020 flow diagram.

During the screening process (see Figure 1), a total of 4,466 documents were excluded. The detailed reasons for exclusion were as follows: the most common reason was a focus on purely technical or engineering materials science (*N* = 2,052)—a necessary exclusion to maintain the review's focus on managerial and policy dimensions rather than device physics; this was followed by subject matter irrelevance (e.g., studies discussing macroeconomic fluctuations but not focusing on industrial policy, *N* = 1,727); and several other categories [including purely market or business analyses (*N* = 277), non-regional policy analyses (*N* = 239), and non-policy-related regional analyses (*N* = 171)]. Following this rigorous screening process, a total of 104 articles were included in this study for narrative synthesis.

### 2.3 Data extraction and synthesis strategy

This study's data analysis employed the Narrative Synthesis method. This approach is widely recognized as a systematic technique for processing and synthesizing heterogeneous research evidence, including diverse theoretical perspectives, research methods, and empirical contexts (Popay et al., 2006; Chiu et al., 2017). It is particularly well-suited for this study's objective, which is not only to “take stock” of what the literature has found, but also to “integrate” these findings to construct a theoretical framework capable of explaining complex policy-ecosystem interactions (Bonevski et al., 2014; Moher et al., 2009).

Given the interpretive nature of this review and the focus on theoretical construction, we did not employ a quantitative scoring tool (like MMAT) to exclude studies. Instead, quality assurance was integrated into the inclusion criteria by: (1) restricting the search to the Web of Science Core Collection to ensure peer-reviewed standards; and (2) performing a full-text appraisal to ensure each included study provided clear research questions and empirical or theoretical evidence relevant to the research objectives. Studies failing to meet these substantive quality thresholds were excluded during the full-text screening phase.

Our synthesis process followed a two-stage analytical framework. The first stage was structured data extraction and coding. We designed a customized data extraction form based on the research questions (RQs) proposed in the introduction. This form was used to systematically extract key information from each article, with primary coding dimensions including basic bibliographic information and core arguments. Crucially, to operationalize the proposed multi-level framework, we coded the data along three theoretical dimensions: (1) Macro-Structure: Categorized by state strategic roles (e.g., Developmental vs. Regulatory State); (2) Meso-Process: Policy instruments were coded using the typology by Borrás and Edquist (Borrás and Edquist, 2013) (regulatory, economic, and soft instruments) to identify governance mechanisms; (3) Micro-Context: Regional attributes were coded based on endowment types (assets vs. institutions). This stage ensured the systematic nature and transparency of the subsequent analysis.

## 3 Results

### 3.1 Descriptive statistics of included studies

To provide a structural overview of the evidence base, Table 2 summarizes the key characteristics of the 104 included articles. The distribution reveals critical trends in current semiconductor policy research.

**Table 2:** Characteristics of Included Studies (*N* = 104).

**Category and sub-category**	**Frequency (*N*)**	**Percentage (%)**
**Panel A: research design**		
Qualitative (empirical)	51	49.0%
Quantitative (empirical)	26	25.0%
Mixed methods	19	18.3%
Review/systematic synthesis	4	3.8%
Theoretical/conceptual	4	3.8%
**Panel B: level of analysis**		
Meso-dominant (policy/governance/industry)	66	63.5%
Balanced multi-level analysis	32	30.8%
Micro-dominant (cluster/firm/technology)	6	5.8%
**Panel C: data source**		
Secondary data/archival	51	49.0%
Primary data (interview/survey)	43	41.3%
Patent/systematic synthesis data	8	7.7%
Not applicable (theory/review)	2	1.9%
**Panel D: geographic focus** ^*^	(Total	
	mentions =	
	200)	
**Core regions**		
China (Mainland)	37	18.5%
Taiwan, Province of China	30	15.0%
United States	19	9.5%
South Korea	18	9.0%
Japan	12	6.0%
United Kingdom	10	5.0%
**Emerging and other regions**		
Europe (aggregated: Germany, France, etc.)^**^	23	11.5%
Southeast Asia (aggregated: Singapore, Malaysia, etc.)^***^	16	8.0%
Others (Brazil, Canada, India, etc.)	15	7.5%
**Panel E: publication trend**		
Recent surge (2023–2025)	48	46.2%
Post-COVID/trade war (2020–2022)	6	5.8%
Pre-2020	50	48.0%

^*^Percentages in Panel D are calculated based on the total number of geographic mentions (*N* = 200) rather than the number of articles, as comparative studies often cover multiple regions.

^**^Europe aggregated includes Germany (6), France (4), Belgium (4), Netherlands (3), and others listed in the Supplementary material.

^***^Southeast Asia aggregated includes Singapore (7), Malaysia (6), Vietnam (3), etc.

First, regarding the level of analysis (Panel B), the literature exhibits a marked polarization. A dominant majority of studies (63.5%) are “meso-dominant,” focusing heavily on specific policy instruments or governance descriptions without systematically linking them to macro-strategies or micro-outcomes. Conversely, only 5.8% of studies are exclusively “micro-dominant.” Crucially, only 30.8% of the sampled literature attempts a “balanced multi-level analysis.” This empirical gap provides strong statistical validation for the “meso-level theoretical gap” identified in the Introduction, underscoring the necessity of the proposed “Dual Fit” framework.

Second, the temporal distribution (Panel E) confirms the timeliness of this review. Research interest shows a significant “recent surge,” with 48 articles published between 2023 and 2025, accounting for 46.2% of the total sample. This trajectory aligns perfectly with the global escalation of “techno-nationalism,” indicating that supply chain resilience has moved from a peripheral topic to a central academic concern.

Third, geographically (Panel D), the research is heavily concentrated on the “Core Nodes” of the global value chain. China's Mainland (18.5% of mentions), Taiwan, Province of China (15.0%), and the United States (9.5%) constitute the primary triangular focus. However, the emergence of studies on Europe (aggregated 11.5%) and Southeast Asia (aggregated 8.0%) reflects the growing trend of supply chain diversification and “friend-shoring.”

### 3.2 The macro-dimension: state strategic roles as institutional structure

The narrative synthesis of the included literature reveals the indisputable central position of the state in semiconductor industry policy, answering this study's first core research question (RQ1). In the context of the SPF framework (Liu and Fan, 2017), these national strategies constitute the “institutional structure”—the static set of rules, legitimacy claims, and resource allocation priorities that define the boundaries of semiconductor governance.

Consistent with the trends observed in the introduction, a large volume of the literature in our sample, particularly articles published after 2018, explicitly defines the semiconductor industry as a strategic arena for national geopolitical and technological sovereignty competition (Manning and Richter, 2023; Kim and Rho, 2024; Hsu, 2025). The literature widely concurs that the motivation for the new wave of policy intervention has undergone a fundamental shift: from the traditional economic logic of correcting “market failure” to a geopolitical logic centered on ensuring “supply chain resilience” and “national security” (Park, 2023; Yuan et al., 2025). This transformation is not merely protectionism but a complex “unbundling” of state motivations, mixing multiple objectives of security, economy, and technological leadership (Rasador and Cunha, 2025). Crucially, this shift redefines the boundary conditions for engineering management, forcing firms to align their operational strategies with national security imperatives rather than pure market efficiency.

Against this macro-backdrop, the evidence in the literature clearly presents three distinct yet overlapping paradigms of the state's strategic role: the Developmental State, the Entrepreneurial State, and the currently dominant Security-Driven State.

#### 3.2.1 The developmental state: the classic paradigm and its new variants

Analyses within the included literature of the rise of the East Asian semiconductor industry commonly adopt the classic “Developmental State” theoretical framework (Hwang and Choung, 2014). In this model, the government is viewed as the “director” and “nurturer” of industrial development. The evidence clearly demonstrates diversified pathways within this model. Case studies on South Korea point out that the government, through a strong bureaucratic system, directed credit, and export-oriented policies, established a close partnership with large conglomerates (Chaebols), which was critical for its memory industry to achieve technological catch-up (Rim et al., 1998; Yang and Yun, 2023; Lee, 2003; Choi, 2014). Analyses of Taiwan, Province of China, offer another classic path: the government established public research institutes (PRIs), namely the Industrial Technology Research Institute (ITRI), to centrally overcome key technological hurdles (such as CMOS processes). It subsequently established leading firms like TSMC through technology diffusion and talent spin-offs, supplemented by cluster cultivation policies at the Hsinchu Science Park (HSIP) (Shyu, 1998; Lee and von Tunzelmann, 2005; Chang et al., 2021; Shiu et al., 2014). Comparative studies confirm that the RIS in Taiwan, Province of China, originated from this “state-led, public R&D” model, whereas the system in Suwon, South Korea, was initially more industry-led (Samsung), albeit with strong subsequent state support (Wong and Lee, 2022).

In the contemporary context, the logic of the developmental state has evolved into new variants within the industrial policy of China's Mainland. The literature indicates that the policy instruments of China's Mainland exhibit stronger characteristics of state capital mobilization, especially through massive capital injections via the National Integrated Circuit Industry Investment Fund (the “Big Fund”) (Meng et al., 2025; Khan, 2024; Tong and Wan, 2023). This model attempts to achieve technological “path creation” through “state logic” rather than “market logic,” but consequently faces risks of path dependency (Meng et al., 2025). Concurrently, the literature reveals the complexities this top-down model faces within a multi-level governance structure. As the central government's macro-strategic intentions are transmitted to the local level, they trigger intense competition among local governments for subsidies and investments. While this competition boosts investment in the short term, it significantly increases the risk of overbuilding and excess capacity in regional industries (Song and Wen, 2023; Baark and Qian, 2025). In this context, the construction of local “state-business-university” tripartite networks is considered a key mechanism for achieving industrial upgrading (Chang et al., 2025).

#### 3.2.2 The entrepreneurial state: market shaping and mission-oriented

The second paradigm is the “entrepreneurial state” (Kuan and West, 2023), where the core role is that of a “market shaper.” Unlike the “guidance” of specific industries by the developmental state, the entrepreneurial state creates and shapes future markets through high-risk, “mission-oriented” public investment at the front end. Analyses in the included literature trace the early success of the US semiconductor industry (Klepper, 2011; Carayannis and Preissler, 2025), noting that large-scale basic research funding and procurement from the Department of Defense (DOD) and the Defense Advanced Research Projects Agency (DARPA) were critical forces driving American technological innovation (del Aguila, 2024; Whetsell et al., 2020). This model emphasizes the state's patient capital investment in basic science and frontier technologies, rather than planning specific commercialization paths.

However, facing the new global competitive landscape, the policy intentions of traditionally market-driven Western economies have also begun to shift, exhibiting hybrid characteristics. The literature points to the US CHIPS and Science Act of 2022 as a prime example of this shift. The Act signifies a major domestic political change from “competition first” to “(domestic) cooperation” to address external challenges (Stokols and Kollar, 2025; Estruth, 2024). On the one hand, the Act aims to attract advanced manufacturing “reshoring” through massive subsidies, bearing strong hallmarks of a developmental state (Park, 2023); on the other hand, its “friend-shoring” initiative is, moreover, a geo-economic strategy serving geopolitical objectives (Park, 2023). Similarly, the “Important Projects of Common European Interest” (IPCEI) launched by the EU to pursue “technological sovereignty” (Konstari and Valkokari, 2025; Lavery and Lopes-Valenca, 2025) is also seen in the literature as a novel policy instrument designed to coordinate member states' resources and reshape Europe's strategic industrial value chains (Lavery and Lopes-Valenca, 2025).

#### 3.2.3 The security-driven state: geopolitics and techno-nationalism

The third paradigm, the “security-driven state,” is central to explaining the new wave of global policy intervention described in the introduction. Among the literature included in this study, empirical research post-2018 overwhelmingly identifies “Techno-nationalism” as the primary driver of current national strategies (Gao et al., 2023; Hsu, 2025; Kim and Rho, 2024; Manning and Richter, 2023). Under this paradigm, the economic attributes of the semiconductor industry have given way to its national security attributes. The core policy objective is no longer simple economic efficiency or market creation, but has shifted to ensuring “supply chain resilience” (Kim and Rho, 2024) and “technological sovereignty” (Lavery and Lopes-Valenca, 2025; Yuan et al., 2025) at any cost.

The synthesis of the literature indicates that this security-oriented macro-intention is profoundly reshaping the industrial policy toolbox and operational decision-making. On the one hand, governments are using massive financial incentives to promote “reshoring” or “friend-shoring” of supply chains to reduce dependence on geopolitical rivals (Liu et al., 2024; Park, 2023). This geopolitical fragmentation is forcing multinational enterprises (MNEs) to make difficult strategic choices between the logic of efficiency and the logic of security (Gao et al., 2023). For instance, recent engineering management research highlights that supplier selection in this era must now integrate complex geopolitical risk parameters alongside traditional cost metrics (Lin et al., 2025). On the other hand, states are increasingly using coercive tools such as export controls, investment screening, and technological decoupling, weaponizing semiconductor supply chain restructuring for geopolitical maneuvering (Baark and Qian, 2025; Gao et al., 2023). This signals that industrial policy is shifting from a traditional “performance-oriented” approach to a “resilience-oriented” one (Kim and Rho, 2024). This fundamental shift from “market-first” to “security-first” constitutes the macro-context (Structure) for understanding the heterogeneity of current global semiconductor policy interventions.

To systematically answer RQ1, we summarize the three state strategic roles and their core characteristics derived from the literature evidence in Table 3.

**Table 3:** Summary and comparison of the state's macro-strategic roles in the literature.

**Strategic role**	**Core motive**	**Key policy mechanisms**	**Representative cases in literature**	**Key literature**
Developmental state	Economic development, technological catch-up, industrial cultivation	State-led industrial planning; Establishing public research institutes (PRIs); directed credit and subsidies; nurturing large leading firms; state capital injection (e.g., “Big Fund”)	South Korea (early Chaebol support); Taiwan, Province of China (ITRI/HSIP model); China's Mainland (“Big Fund” model)	(Baark and Qian, 2025; Chang et al., 2021, 2025; Choi, 2014; Huang and Malkin, 2025; Khan, 2024; Lee, 2003; Meng et al., 2025; Rim et al., 1998; Shiu et al., 2014; Song and Wen, 2023; Tong and Wan, 2023; Wong and Lee, 2022; Yang and Yun, 2023)
Entrepreneurial state	Market creation, mission-oriented innovation, solving grand challenges	Front-end basic research funding (e.g., DARPA); public procurement; public-private partnerships (PPP); mission-oriented programs (e.g., IPCEI).	United States (early DOD/DARPA funding); European Union (IPCEI projects)	(Carayannis and Preissler, 2025; del Aguila, 2024; Estruth, 2024; Klepper, 2011; Konstari and Valkokari, 2025; Kuan and West, 2023; Lavery and Lopes-Valenca, 2025; Spencer and Grindley, 1993; Stokols and Kollar, 2025; Whetsell et al., 2020)
Security-driven state	National security, geopolitical competition, techno-nationalism	Ensuring supply chain resilience; massive subsidies (e.g., CHIPS Act); “reshoring” and “friend-shoring”; export controls and technological decoupling; motive unbundling (security/economic/technological)	Contemporary US (CHIPS Act, export controls); contemporary EU (technological sovereignty); contemporary China's Mainland (self-reliance and controllability)	(Gao et al., 2023; Hsu, 2025; Kim and Rho, 2024; Konstari and Valkokari, 2025; Lin et al., 2025; Liu et al., 2024; Manning and Richter, 2023; Park, 2023; Rasador and Cunha, 2025; Stokols and Kollar, 2025; Yuan et al., 2025)

### 3.3 The meso-dimension: governance mechanisms as the dynamic process

If the macro-level national strategy (RQ1) discussed in section 3.2 sets the “structure” of policy, then the meso-dimension (RQ2) represents the “dynamic process”—the pathways through which strategic intentions are translated into industrial reality. According to the SPF perspective, structure alone cannot guarantee function; it requires a robust “process” of governance to mediate resource flows and align incentives (Liu and Fan, 2017). The Narrative Synthesis of the included literature indicates that analysis at this meso-implementation level can be divided into two sub-topics: the selection of policy instruments (what) and the governance mechanisms (how).

#### 3.3.1 The selection and combination of policy instruments

The included evidence base reveals an extremely complex Policy Toolbox, through which governments (and regions) combine various instruments to guide the development of the regional semiconductor industry. A deep synthesis of this literature demonstrates that, given the dually capital- and knowledge-intensive nature of the semiconductor industry, the effectiveness of policy instruments depends more on their functional “specificity” and the way they are combined. We categorize the instruments discussed in the literature into the following five functional combinations:

First, R&D and innovation instruments. Given the industry's position at the technological frontier, PRIs and government-funded R&D consortia are critical. Recent scholarship in IEEE-TEM argues that for planetary survival and critical transitions, “technology-forcing” mechanisms—where regulators mandate performance standards beyond current capabilities—are more effective than technology-neutral subsidies (Phillips and Pham Thi, 2024). While ITRI in Taiwan (China) (Chang et al., 2021; Chang and Tsai, 2002; Lee and von Tunzelmann, 2005; Shyu, 1998; Wong and Lee, 2022) represents a classic “catch-up” R&D model, contemporary instruments increasingly resemble this “forcing” logic to accelerate sovereignty goals. The US DARPA model represents another “mission-oriented” approach to front-end basic research funding (Kuan and West, 2023), while the US SEMATECH consortium serves as another paradigm of government-industry cooperation (Spencer and Grindley, 1993). Europe's transnational R&D platforms (e.g., IMEC in Belgium) are its core instrument for maintaining an edge in specific fields (like EUV lithography) (Huggins et al., 2023). Japan's “Very Large-Scale Integration” (VLSI) joint R&D project in the 1970s is considered an early successful model of government-coordinated R&D consortia (Chiang, 2000; Lynn, 1998; Wang et al., 2024).

Second, capital and financial instruments. The massive capital expenditure (CapEx) required for semiconductor manufacturing makes it a focal point for financial incentives. The literature's discussion of such instruments is particularly concentrated, especially regarding the new wave of policy interventions. This includes massive direct subsidies and investment tax credits, as exemplified by the US CHIPS Act (Estruth, 2024; Kollar, 2025; Stokols and Kollar, 2025), as well as the direct state capital equity investment represented by the National Integrated Circuit Industry Investment Fund (the “Big Fund”) in China's Mainland (Chang et al., 2025; Liu, 2024; Tong and Wan, 2023). Concurrently, the literature notes that the effectiveness of these instruments is highly controversial. For instance, research on China's Mainland indicates that national subsidies have a limited positive impact on firm productivity and indigenous innovation (Huang and Malkin, 2025; Song and Wen, 2023), and the excessive subsidies provided by local governments competing for investment have led to significant risks of overcapacity (Song and Wen, 2023).

Third, cluster and infrastructure instruments. The semiconductor industry exhibits a high degree of geographical agglomeration, making policy instruments for cultivating industrial clusters crucial. The analysis of the HSIP in Taiwan, Province of China, is a typical example, demonstrating how authorities attract and nurture firm ecosystems by providing high-quality infrastructure, tax incentives, and “one-stop” services (Chang and Tsai, 2002; Shyu, 1998; Wong and Lee, 2022). European experiences are more diverse; for example, the “MINATEC” micro and nanotechnology innovation center in Grenoble, France, is a successful case of local government, universities, and firms jointly building and sharing R&D infrastructure (Johnston and Huggins, 2023). However, the literature also documents failures of such instruments. For example, the attempt by North East England to attract investment through “flagship projects,” such as building advanced fabs, failed rapidly after the multinational corporation (Siemens) divested. It failed to generate sustainable regional development due to a lack of supporting policies and local embeddedness (Kirchner, 2000).

Fourth, talent cultivation and attraction instruments. Semiconductor competition is, in essence, competition for high-skilled talent. Therefore, policy instruments designed to cultivate and attract talent are repeatedly mentioned. These include national-level talent introduction programs, special funding for university research, and mechanisms promoting the formation of “industry-university-research” (IUR) networks (Chen and Wang, 2023; Fauth et al., 2024; Rasiah et al., 2016a). The literature suggests that the success of a RIS depends heavily on its ability to attract and retain high-skilled engineers and scientists (Edgington and Hayter, 2013; Lin and Rasiah, 2014). The case of Singapore illustrates another path, where, through proactive immigration policies and incentives for MNE R&D centers, it successfully established itself as a global hub for semiconductor talent (Mathews, 1999).

Fifth, market, trade, and regulatory instruments. These instruments have become particularly prominent under the “Security-Driven State” paradigm. Literature analysis shows that governments are increasingly “weaponizing” market access and regulatory rules (Aoyama et al., 2024; Germann et al., 2024). This not only includes export controls (Baark and Qian, 2025; Gao et al., 2023; Liu and Lin, 2025) but also the strategic use of intellectual property (IP) and standards. For instance, the “watchful waiting” strategy in standard-essential patents (SEPs) for 5G technology highlights how firms strategically delay IP declarations to navigate regulatory uncertainty (Herzberg et al., 2024). Similarly, investment screening and “friend-shoring” initiatives (Aoyama et al., 2024; Estruth, 2024) represent a shift from market-enabling to market-restricting governance.

To systematically answer the “instruments” part of RQ2, we summarize the five functional policy instruments discussed above in Table 4.

**Table 4:** Functional categorization of semiconductor industry policy instruments in the literature.

**Tool function category**	**Policy objective**	**Specific tool cases in literature**	**Key representative literature**
R&D and innovation	Knowledge creation/diffusion, technological breakthrough	Public Research Institutes (PRIs) (e.g., ITRI in Taiwan, Province of China; EU IMEC); mission-oriented funding (e.g., US DARPA); technology forcing mechanisms; R&D consortia (e.g., Japan's VLSI project)	(Chang et al., 2021; Chang and Tsai, 2002; Chiang, 2000; Huggins et al., 2023; Kuan and West, 2023; Lee and von Tunzelmann, 2005; Lynn, 1998; Phillips and Pham Thi, 2024; Shyu, 1998; Spencer and Grindley, 1993; Wang et al., 2024)
Capital and financial	Reduce capital costs, guide investment	Direct subsidies (e.g., US CHIPS Act); state fund equity investment (e.g., “Big Fund” in China's Mainland); investment tax credits	(Estruth, 2024; Huang and Malkin, 2025; Kollar, 2025; Liu, 2024; Meng et al., 2025; Song and Wen, 2023; Stokols and Kollar, 2025; Tong and Wan, 2023)
Cluster and infrastructure	Nurture industrial ecosystems, reduce transaction costs	Science parks / industrial parks (e.g., HSIP in Taiwan, Province of China; France's MINATEC); flagship projects (e.g., North East England case)	(Chang and Tsai, 2002; Johnston and Huggins, 2023; Kirchner, 2000; Shyu, 1998; Wong and Lee, 2022)
Talent cultivation and attraction	Ensure supply of high-skilled labor	National talent programs; funding for industry-university-research (IUR) networks; immigration and talent hub policies (e.g., Singapore)	(Chen and Wang, 2023; Edgington and Hayter, 2013; Fauth et al., 2024; Lin and Rasiah, 2014; Mathews, 1999; Rasiah et al., 2016a)
Market, trade, and regulatory	Secure markets/reconstruct supply chains	Export controls; investment screening; Standard-essential patent strategies; “Friend-shoring” initiatives; government unified procurement (early); foreign subsidies regulation (FSR)	(Aoyama et al., 2024; Baark and Qian, 2025; Estruth, 2024; Gao et al., 2023; Germann et al., 2024; Herzberg et al., 2024; Liu and Lin, 2025; Ruck, 2024)

#### 3.3.2 The coordination and evolution of governance mechanisms

The second core issue of the meso-dimension is governance mechanisms. In the SPF framework, this represents the “steering” function of the process. The literature analysis indicates that the failure of macro-strategic intentions often lies not in the choice of tools (what) but in governance failure (how). We distill two core governance challenges: multi-level coordination and public-private orchestration.

First, multi-level governance (MLG) and central-local relations are key recurring themes in the literature. In federal or large unitary states, the central government's macro-strategy must be executed by local governments, which frequently creates coordination dilemmas. The analysis of China's Mainland is particularly salient, where the literature notes that the central “big fund” and industrial planning intentions have triggered fierce “tournament-style competition” at the local level (Khan, 2024; Song and Wen, 2023). Local governments, vying for projects and subsidies, have caused severe redundant construction and resource misallocation (Lin and Rasiah, 2014; Song and Wen, 2023; Tong and Wan, 2023). Analysis of the US also points out that although the CHIPS Act is federal, states (e.g., Arizona, New York) are also engaged in fierce subsidy competition to attract giants like TSMC and Intel (Germann et al., 2024; Kollar, 2025; Stokols and Kollar, 2025). In the EU, this MLG is embodied in the strategic game between the European Commission and member states (such as Germany and France); the IPCEI project is itself a mechanismal innovation attempting to coordinate transnational resources within this complex governance structure (Huggins et al., 2023; Johnston and Huggins, 2023; Konstari and Valkokari, 2025).

Second, the effectiveness of Public-Private Coordination Networks is another core aspect of meso-level governance. The literature contrasts different coordination models. The ITRI model in Taiwan, Province of China, represents the “PRI-mediated” model, where authorities use a semi-public entity to coordinate R&D, talent mobility, and enterprise incubation (Chang et al., 2021; Chang and Tsai, 2002; Lee and von Tunzelmann, 2005; Shyu, 1998). South Korea's model relies more on tight coordination between the government and a few large Chaebols (like Samsung) (Choi, 2014; Lee, 2003; Lin and Rasiah, 2014; Yang and Yun, 2023). Crucially, effective governance requires an “orchestrator” capable of managing network complexity. As Noviaristanti et al. (2024) demonstrate in their study of accelerator networks, orchestration activities—such as knowledge mobility and network stability—are essential for value creation (Noviaristanti et al., 2024). In China's Mainland, the literature observes the formation of a “state-business-university” tripartite network, the coordinative efficacy of which directly impacts the success or failure of regional industrial upgrading (Chen and Wang, 2023). The case of Singapore offers a governance model of “Institutional Learning,” where government agencies (like the Economic Development Board, EDB) dynamically adjust their policy instruments and governance methods through long-term interaction and “co-evolution” with MNEs to achieve strategic industrial upgrading (Mathews, 1999). The literature widely agrees that the lack of an effective ecosystem orchestrator (Germann et al., 2024; Kirchner, 2000) is the main reason for the disconnect between policy intentions and regional realities, ultimately leading to policy failure.

### 3.4 The micro-dimension: regional ecosystems as contextual structure

The previous sections elaborated on the macro-strategy (structure) and meso-governance (process). However, the success of policy ultimately depends on the “contextual structure” of the target region (RQ3). Top-down interventions are not enacted in a vacuum but are embedded within specific “structural endowments” that constrain or enable agency. The narrative synthesis indicates that the existing characteristics of the RIS are a key moderating variable. We deconstruct these characteristics into “static endowments” and “dynamic capabilities.”

#### 3.4.1 Static endowments of the regional innovation ecosystem

Static endowments refer to the assets, resources, and institutional structures that a region inherits or accumulates prior to policy intervention, which determine the “starting point” for the intervention.

First, industrial and technological assets are the “hardware” of the regional ecosystem. This includes not only physical infrastructure but, more importantly, existing firm networks, technological foundations, and human capital stock. Comparative cases in the literature clearly illustrate this point. The success of the HSIP in Taiwan, Province of China, was largely built upon the semiconductor manufacturing technology base accumulated in its early days through ITRI and United Microelectronics Corporation (UMC) (Chang et al., 2021; Lee and von Tunzelmann, 2005; Shiu et al., 2014; Shyu, 1998). The ecosystem in Suwon, South Korea, was dominated by Samsung, a “hub-and-spoke” core enterprise that served as the most critical regional asset (Choi, 2014; Lee, 2003; Wong and Lee, 2022). The case of “Silicon Saxony” in Germany demonstrates another asset base; the region's success was built upon the deep “industrial heritage” and skilled worker base left over from the microelectronics industry of the German Democratic Republic (GDR) period (Rim et al., 1998). The literature also emphasizes the importance of high-level universities and research institutions as regional assets, such as the MINATEC innovation center in the Grenoble region of France, which was built around world-class micro- and nanotechnology research institutions (Johnston and Huggins, 2023).

However, the literature also reveals the “reverse side” of asset endowments, namely the inertia or vulnerability that “industrial heritage” can bring. This reflects the broader mechanism of path dependence in industrial development, where early technological choices and asset accumulation can create self-reinforcing lock-in effects (He et al., 2024). The case of North East England, a region of traditional industrial decline, is particularly profound. The region attempted to achieve an industrial leap by introducing a “flagship project”—a Siemens fab—but the policy failed when Siemens divested (Dawley, 2007). This indicates that merely injecting a single “flagship asset” is insufficient to change the fundamental structure of a region. Interestingly, however, this study also found that the world-class fab and experienced management team left behind by this failed investment, as a “dormant” asset, provided a unique endowment for subsequently attracting investment from Atmel (Dawley, 2007). This illustrates that the value of regional assets is dynamic and highly “place-specific.” Similarly, in China's Mainland, the literature points to vast differences in asset endowments across regions: regions like Shanghai and Beijing possess deep assets in universities and research institutions (Khan, 2024; Zhang, 2023), whereas many latecomer regions rely more on inter-local competition to attract “flagship project” investments, which exacerbates the risk of overbuilding (Baark and Qian, 2025; Song and Wen, 2023).

Second, institutional assets are the “software” of the regional ecosystem—that is, the local institutions, norms, and trust networks that support innovation activities, often referred to as “institutional thickness.” The literature repeatedly emphasizes this point. The success of Hsinchu, Taiwan, Province of China, was not just due to the tangible science park, but more so to ITRI, as a key “intermediary organization,” facilitating technology diffusion, talent mobility, and informal knowledge spillovers among firms, thus forming an efficient institutional environment (Chang and Tsai, 2002; Hu, 2011; Lee and von Tunzelmann, 2005; Shyu, 1998). The literature refers to institutions like ITRI as “revolutionary peripheral agencies,” which are institutionally detached from rigid bureaucracies and short-sighted market pressures, enabling them to execute high-risk, long-cycle innovation tasks (Hsu, 2004). In Europe, the literature notes that the success of regional innovation policy relies on a robust institutional network jointly involving Small and Medium-sized Enterprises (SMEs), research institutions, and local government (Johnston and Huggins, 2023). A comparative study of Saxony, Germany, and South Korea also found that while both emphasized institutional support, Saxony focused more on nurturing SME networks, whereas South Korea focused more on supporting large enterprises (Yang and Yun, 2023). Conversely, in some regions of China's Mainland, despite massive material and capital investment, the literature indicates that local protectionism and “relationship-based” governance can sometimes hinder the formation of a unified, open market-based institutional environment (Song and Wen, 2023; Tong and Wan, 2023).

#### 3.4.2 Dynamic capabilities of the regional innovation ecosystem

If static endowments determine the “starting point,” then dynamic capabilities determine whether a region can leverage these endowments to “evolve.” Dynamic capabilities refer to the “agency” of an RIS to adapt to external changes, absorb external knowledge, and achieve path upgrading. The synthesis of the literature points to three key capabilities.

First is the knowledge absorption and transformation capability. That is, a region must not only possess universities and research institutions (static endowments) but must also have the capability to transform this scientific knowledge into commercial innovation. The literature indicates that the tightness of IUR networks within the region is key (Rasiah et al., 2016a). This collaborative dynamic is increasingly analyzed through the lens of the “Quadruple Helix” model, where platform-based firms drive innovation by aligning university, industry, and government actors (Wang, 2024). For example, literature analyzing the factors determining regional development differences in the semiconductor industry of Taiwan, Province of China, found that the stock of high-skilled talent and local firms' R&D investment were core determinants (Lin and Rasiah, 2014). Research on China's Mainland also found that the coordination efficiency of the local government-led “state-business-university” tripartite networks directly determines whether the region can achieve industrial upgrading (Fauth et al., 2024; Zhang, 2023). The formation of this capability involves multiple mechanisms: it can be through the firm itself (e.g., Samsung) achieving rapid technological learning and “catch-up” via sustained R&D investment and technology acquisition (Choi, 2014; Lee, 2003; Rim et al., 1998); it can also be through government-coordinated R&D consortia [e.g., Japan's VLSI project (Wang et al., 2024) or the US SEMATECH (Spencer and Grindley, 1993)] to elevate the entire region's industry technology level (Chiang, 2000); ultimately, this capability is embodied in the “upgrading” of the RIS, where knowledge sources shift from a reliance on foreign sources to localization and become increasingly linked with science (Wong and Lee, 2022).

Second is the strategic coupling and co-evolution capability. This refers to the capability of a regional ecosystem to effectively interface its local assets with Global Value Chains (GVCs). The case of Singapore is the best illustration. The literature notes that Singapore's semiconductor industry was not indigenously cultivated; rather, through the EDB, a highly efficient governance agency, it proactively embedded its position within the global strategies of MNEs (Mathews, 1999). More importantly, Singapore's governance agencies demonstrated an “institutional learning” capability; they “co-evolved” with MNEs, dynamically adjusting local policies and services, thereby driving the local industry's strategic upgrading from low-end assembly and testing to high-end R&D and design (Mathews, 1999). This “coupling” capability manifests differently in various contexts: North East England demonstrated a “recoupling,” leveraging the “dormant assets” left by the failed Siemens investment to successfully couple with the global expansion strategy of Atmel (Dawley, 2007). In the current geopolitical context, the literature notes that US states (like Arizona) are attempting, through the CHIPS Act subsidies, a new form of “strategic coupling” with leading firms like TSMC, one that is led by government incentives (Kollar, 2025; Stokols and Kollar, 2025).

Third is path dependency and evolutionary resilience. A region's evolutionary path is deeply influenced by its initial endowments, i.e., “path dependency.” Comparative studies in the literature clearly show this: Hsinchu, Taiwan, Province of China, originated from a “public R&D-led” model (Chang et al., 2021; Chang and Tsai, 2002) and evolved into a “Marshallian” innovation system with a relatively dispersed firm network and active SMEs; Suwon, South Korea, originated from a “firm-led” model (Choi, 2014; Lee, 2003) and evolved into a “hub-and-spoke” system, with innovation highly concentrated in Samsung (Wong and Lee, 2022). The revival of Germany's “Silicon Saxony” is also highly dependent on its microelectronics path from the GDR era (Johnston and Huggins, 2023). However, the other side of path dependency is the risk of “lock-in.” Analyses of the “Big Fund” model in China's Mainland implicitly contain concerns that “state logic” might override “market logic,” thus causing the regional industry to become “locked-in” to specific technologies or models (Khan, 2024; Meng et al., 2025; Tong and Wan, 2023).

Therefore, a region's “resilience”—its ability to cope with shocks and achieve “path unlocking”—becomes particularly important. Empirical configuration analysis suggests that such resilience is not guaranteed by endowments alone but requires specific combinations of marketization levels and innovation support (Chen et al., 2024). The case of North East England demonstrates another kind of “resilience”: the region utilized the “heritage” from a failed investment (Dawley, 2007) and relied on the agency of local institutions (Dawley, 2007) to successfully achieve path renewal. The literature further points out that the key dynamic capability of “peripheral agencies” like ITRI (Taiwan, Province of China) or SITRA (Finland) is precisely their ability to initiate radical innovations, helping the nation and region break free from existing industrial path dependencies (Hu, 2011).

To systematically answer RQ3, we summarize the key dimensions of the micro-context derived from the literature evidence in Table 5.

**Table 5:** Dimensional categorization of the regional innovation ecosystem (micro-context) in the literature.

**Dimension**	**Key concept**	**Key findings and cases in literature**	**Key representative literature**
Static endowments	Industrial/tech assets	A region's existing firm base [e.g., SK/Suwon (Choi, 2014; Lee, 2003; Wong and Lee, 2022)], technological heritage [e.g., GER/Saxony (Johnston and Huggins, 2023; Rim et al., 1998)], public R&D facilities [e.g., FR/Grenoble (Johnston and Huggins, 2023)], human capital stock (Lin and Rasiah, 2014), Path dependence risks (He et al., 2024) and “dormant assets” [e.g., UK/North East (Dawley, 2007)] form the basis for intervention.	(Chang et al., 2021; Choi, 2014; Dawley, 2007; He et al., 2024; Johnston and Huggins, 2023; Kim and Rho, 2024; Lee, 2003; Lee and von Tunzelmann, 2005; Lin and Rasiah, 2014; Rim et al., 1998; Shiu et al., 2014; Wong and Lee, 2022)
	Institutional assets	Efficient “intermediary/peripheral agencies” [e.g., ITRI in Taiwan, Province of China (Chang et al., 2021; Chang and Tsai, 2002; Hsu, 2004; Hu, 2011; Shyu, 1998)], IUR networks [e.g., China's Mainland (Khan, 2024; Zhang, 2023)], and local “institutional thickness” (e.g., GER/Saxony vs. SK [Yang and Yun, 2023); Europe (Johnston and Huggins, 2023)]. Institutional deficits [e.g., local protectionism (Song and Wen, 2023; Tong and Wan, 2023)] constitute obstacles.	(Chang and Tsai, 2002; Hsu, 2004; Hu, 2011; Johnston and Huggins, 2023; Khan, 2024; Lee and von Tunzelmann, 2005; Shyu, 1998; Song and Wen, 2023; Tong and Wan, 2023; Yang and Yun, 2023; Zhang, 2023)
Dynamic capabilities	Absorptive capacity	The capability of a region to translate scientific knowledge into commercial innovation relies on highly skilled talent (Lin and Rasiah, 2014), corporate R&D investment [e.g., South Korea/Samsung (Rim et al., 1998; Lee, 2003)], R&D consortia [e.g., Japan/VLSI (Chiang, 2000; Wang et al., 2024)] or the US/SEMATECH (Spencer and Grindley, 1993), and network coordination efficiency (Fauth et al., 2024; Zhang, 2023). Ultimately, this manifests as the “upgrading” of the RIS (Wong and Lee, 2022).	(Chiang, 2000; Fauth et al., 2024; Lee, 2003; Lin and Rasiah, 2014; Rasiah et al., 2016b; Rim et al., 1998; Spencer and Grindley, 1993; Wang, 2024; Wang et al., 2024; Wong and Lee, 2022; Zhang, 2023)
	Strategic coupling	The region's ability to “co-evolve” its local assets with GVCs [e.g., Singapore EDB model (Mathews, 1999)]; or “recouple” using “dormant assets” [e.g., UK/North East (Dawley, 2007)]; or government incentive-led coupling [e.g., US/Arizona (Kollar, 2025; Stokols and Kollar, 2025)].	(Dawley, 2007; Kollar, 2025; Mathews, 1999; Stokols and Kollar, 2025)
	Path evolution and resilience	A region's evolution is subject to “path dependency” from initial endowments [e.g., Hsinchu vs. Suwon (Chang et al., 2021; Lee, 2003; Shiu et al., 2014; Wong and Lee, 2022)]. State logic may lead to “lock-in” (Khan, 2024; Meng et al., 2025; Tong and Wan, 2023). Regional “resilience” is shown by leveraging “failure heritage” (Dawley, 2007) or achieving “path unlocking” (Dawley, 2007) via “peripheral agencies” (Hsu, 2004). Resilience configuration (Chen et al., 2024).	(Chang et al., 2021; Chen et al., 2024; Dawley, 2007; Hsu, 2004; Johnston and Huggins, 2023; Khan, 2024; Lee, 2003; Meng et al., 2025; Shiu et al., 2014; Tong and Wan, 2023; Wong and Lee, 2022)

## 4 Discussion

### 4.1 Synthesis of core themes: the structure, process, and context

The core question of this study originates from an empirical observation (see section 1): In the new wave of global semiconductor industry policy, why do similar macro-policy intentions produce starkly divergent industrial outcomes in different regions? To answer this question, the section 3, following this study's multi-level analytical framework, conducted a systematic narrative synthesis of the 104 included core articles and related supplementary literature. They sequentially deconstructed the macro-level state's strategic role (RQ1), the meso-level policy instruments and governance mechanisms (RQ2), and the micro-level RIS and contexts (RQ3). However, an independent analysis of these three dimensions is insufficient to answer the study's core question. The literature evidence repeatedly indicates that policy success or failure depends not on any single-level “optimal design,” but on whether dynamic “fit” or “alignment” can be achieved among these three levels. Therefore, the core task of this section (to answer RQ4) is to integrate the analytical evidence from the previous three sections into a unified mechanistic framework from an “interactive” rather than “isolated” perspective. To this end, drawing on Mertonian Middle-Range Theory (Abner et al., 2017), we adopt a SPF perspective to synthesize these findings. Unlike static structuralism, the SPF framework posits that the “structure” (macro-strategy and micro-endowments) can only generate the desired “function” (resilience) through a dynamic “process” of governance (Liu and Fan, 2017).

#### 4.1.1 The macro-dimension as institutional structure: from “development” to “security”

The analysis in section 3 demonstrated that the state's macro-strategic role in semiconductor policy has undergone a profound evolution, constituting a shift in the “Institutional Structure” of the industry. The classic “Developmental State” paradigm, whether South Korea's Chaebol coordination model (Choi, 2014; Hwang and Choung, 2014; Lee, 2003) or Taiwan, Province of China's ITRI-mediated model (Chang et al., 2021; Lee and von Tunzelmann, 2005; Shiu et al., 2014; Shyu, 1998), had economic catch-up as its core motive. The “Entrepreneurial State” paradigm focused on “market shaping” through DARPA-style mission-oriented investments (Kuan and West, 2023; Spencer and Grindley, 1993). However, the overwhelming consensus in the literature (especially post-2018) is that the primary driver for current policy intervention has shifted to the “Security-Driven State” (Hsu, 2017; Manning and Richter, 2023; Park, 2023). The essence of this shift is that considerations of “Techno-nationalism” (Park, 2023) and “Supply Chain Resilience” (Lu and Moorthy, 2025; Zhou et al., 2024) have overridden traditional economic efficiency logic. The core implication of this structural finding is: the “objective function” of contemporary semiconductor policy has fundamentally changed, shifting from the pursuit of “efficiency” to the pursuit of “security,” “redundancy,” and “control” (Aoyama et al., 2024; Yuan et al., 2025). This strategic turn, which prioritizes ensuring supply chain “autonomy and controllability” (Baark and Qian, 2025; Hwang and Choung, 2014; Park, 2023), consequently places starkly different demands on its meso-level implementation logic.

#### 4.1.2 The meso-dimension as governance process: the centrality of coordination

The analysis in section 3 revealed that the “policy toolbox” itself is relatively mature, with five major categories of instruments (R&D, capital, cluster, talent, regulatory) appearing repeatedly in the literature (Chen and Wang, 2023; Chiang, 2000; Lee and von Tunzelmann, 2005; Meng et al., 2025; Spencer and Grindley, 1993; Tong and Wan, 2023). However, the evidence also clearly indicates that policy failure often lies not in what tools are used (What), but in how they are implemented (How). The core challenge at the meso-level is “Governance Failure,” which in the SPF framework represents a breakdown in the “governance process.” This challenge is particularly prominent in two dimensions: First, the coordination dilemma of MLG. Central macro-intentions (e.g., China's Mainland's “Big Fund”) are easily distorted into “tournament-style competition” during local implementation, leading to severe redundant construction and resource misallocation (Meng et al., 2025; Song and Wen, 2023; Tong and Wan, 2023). Supplementary literature corroborates this, noting that regions significantly “adjust and deviate” (Brueck et al., 2025; Zhang et al., 2023) when executing national policies, making strategic alignment between central and local governments difficult to guarantee. Second, the vulnerability of Public-Private Coordination Networks. Effective policy implementation relies on a coordination mechanism that possesses high-level expertise, mutual trust, and is embedded in the industry. The lack of an effective “ecosystem orchestrator” often leads to a disconnect between policy intent and operational reality (Chen and Ling, 2024). The efficient coordination by Singapore's EDB is seen as a paradigm (Mathews, 1999), while Taiwan, Province of China's ITRI played a key “intermediary” role (Chang and Tsai, 2002; Shyu, 1998). Conversely, as the North East England case shows (Kirchner, 2000), although local authorities successfully attracted a “flagship project,” the policy failed upon divestment due to the lack of an effective, locally embedded public-private network to absorb and upgrade the project. The core implication of this meso-finding is: The “meso-level” is the “converter” and “filter” for macro-strategic intentions, and the failure of governance mechanisms (especially central-local and public-private relations) is the primary cause of policy distortion and failure to meet objectives.

#### 4.1.3 The micro-dimension as contextual structure: endowments and capabilities

The analysis in section 3 confirms that the RIS, as the “landing point” for policy intervention, constitutes the “contextual structure” that constrains policy agency. The literature evidence strongly suggests that policy effectiveness is highly “context-dependent.” This context is determined by two levels: First, the “path dependency” of static endowments. The “hardware” a region inherits [e.g., the industrial heritage of Saxony, Germany (Johnston and Huggins, 2023) or the research facilities of Grenoble, France (Huggins et al., 2023)] and the “software” [e.g., the institutional thickness of Hsinchu, Taiwan, Province of China (Chang and Tsai, 2002; Lee and von Tunzelmann, 2005; Shyu, 1998)] constitute an insurmountable “starting point” for policy intervention. Policy must operate within the constraints of these existing endowments; for example, the institutional support paths of South Korea and Saxony, Germany, were starkly different due to their different endowments (Yang and Yun, 2023). Second, the “evolutionary potential” of dynamic capabilities. Regions are not passive recipients; their “agency”—comprising absorptive capacity (Fauth et al., 2024; Lin and Rasiah, 2014), strategic coupling capability [e.g., Singapore (Carayannis and Preissler, 2025)], and evolutionary resilience [e.g., North East England's reuse of failure heritage (Dawley, 2007; Kirchner, 2000)]—is the key determinant of whether a region can leverage policy opportunities to achieve “path unlocking.” Broader regional theories also support this view, namely that development models differ greatly across regions (Bhattacharya et al., 2017; Rolf et al., 2025). The core implication of this micro-finding is: The regional context is both a “constraint” on policy intervention (static endowments) and an “agentic partner” in policy success (dynamic capabilities).

### 4.2 Constructing the “dual fit” middle-range theory

**Figure 2:**
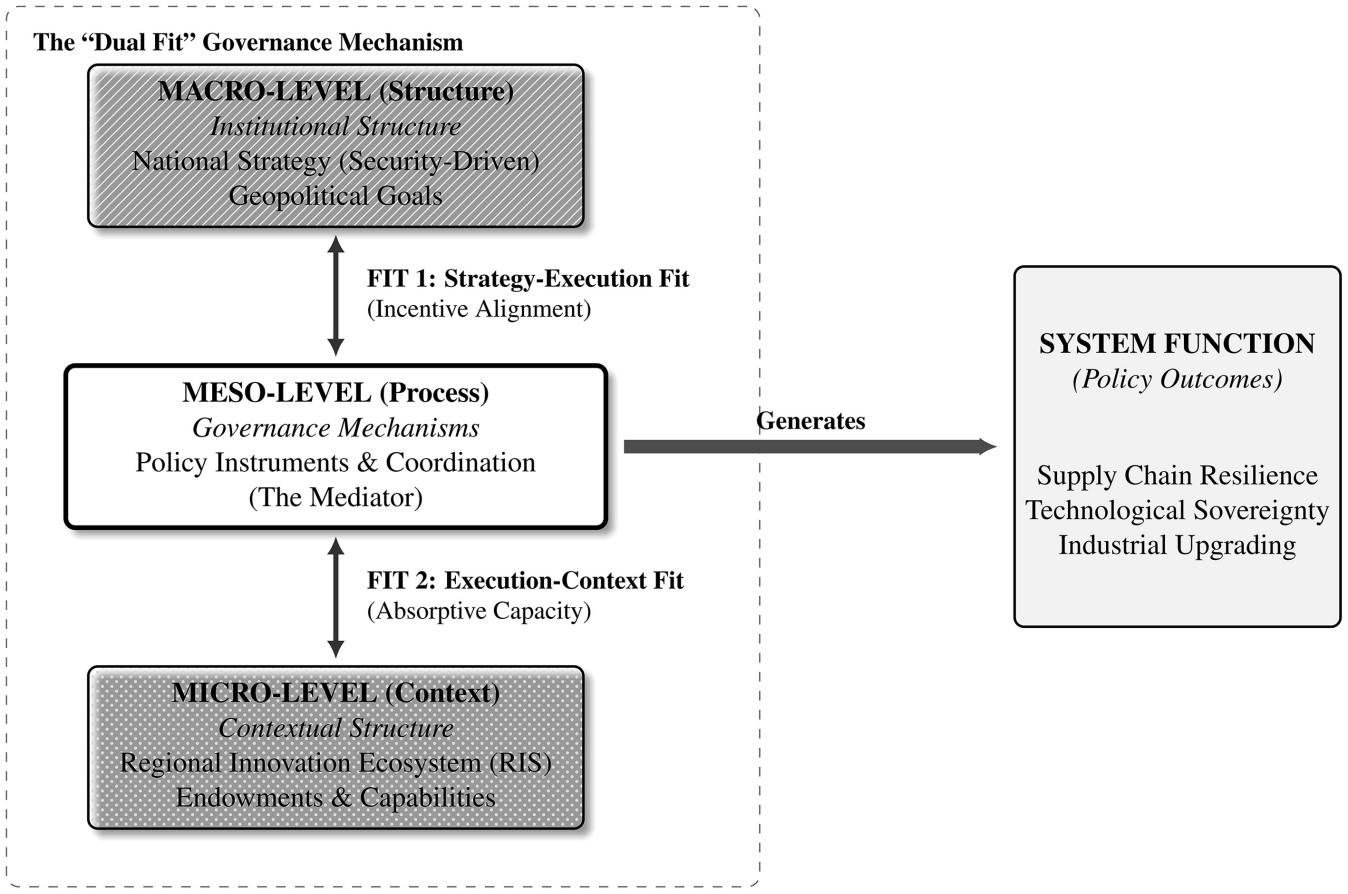
The “dual fit” governance mechanism.

The synthesis in Table 6 clearly indicates that the heterogeneous outcomes of policy stem from complex interactions among the macro, meso, and micro levels. No “optimal” design at any single level can guarantee policy success. A “security-driven” macro-strategy (RQ1) that encounters “governance failure” in its meso-level implementation (RQ2) while ignoring the “context-dependent” micro-level reality (RQ3) is highly prone to implementation failure. For example, the literature's analysis of local government competition in China's Mainland (Khan, 2024; Meng et al., 2025; Song and Wen, 2023; Tong and Wan, 2023) is a typical manifestation of macro-intent (RQ1) being distorted at the meso-level (RQ2) due to poor central-local coordination. Similarly, the North East England case (Kirchner, 2000) illustrates that even with a national-level “flagship project” tool (RQ2), if the micro-region (RQ3) lacks the necessary institutional thickness and dynamic capabilities to “embed” and “digest” this investment, the policy faces a high risk of ultimate fail. Therefore, the literature evidence collectively points to a conclusive argument that transcends single-level analysis: policy effectiveness is a problem of Mechanism Fit. As noted in related research, achieving sustainable development goals requires “Institutional Alignment” (Healy and Debski, 2017; Ouyang, 2006). Based on this, this study proposes a comprehensive “dual fit” framework as a middle-range theory to explain these complex interactions (see Figure 2).

**Table 6:** Synthesis of key themes and representative literature across multi-level dimensions.

**Analytical dimension**	**Research questions (RQs)**	**Core themes in literature**	**Key findings from synthesis**	**Key representative literature**
Macro	(RQ1) What is the strategic role of the state?	Developmental state Entrepreneurial state Security-driven state	The core motive for policy has shifted from “economic/market” to “security/resilience” (Hsu, 2017; Park, 2023), causing the overall policy goal to shift from “efficiency” to “control” (Aoyama et al., 2024; Lu and Moorthy, 2025; Yuan et al., 2025; Zhou et al., 2024).	(Aoyama et al., 2024; Baark and Qian, 2025; Carayannis and Preissler, 2025; Chang et al., 2021; Choi, 2014; del Aguila, 2024; Estruth, 2024; Hsu, 2017; Hwang and Choung, 2014; Khan, 2024; Klepper, 2011; Konstari and Valkokari, 2025; Kuan and West, 2023; Lee, 2003; Lee and von Tunzelmann, 2005; Lu and Moorthy, 2025; Manning and Richter, 2023; Park, 2023; Shiu et al., 2014; Shyu, 1998; Spencer and Grindley, 1993; Stokols and Kollar, 2025; Tong and Wan, 2023; Yuan et al., 2025; Zhou et al., 2024)
Meso	(RQ2) How do policy instruments and governance mechanisms operate?	Policy toolbox (Chen and Wang, 2023; Chiang, 2000; Meng et al., 2025) Multi-level governance (MLG) coordination Public-private (P-P) coordination networks	The core of policy failure is often “Governance Failure,” not “instrument failure.” Misalignment of central-local interests (Brueck et al., 2025; Meng et al., 2025; Song and Wen, 2023; Zhang et al., 2023) and the absence of P-P coordination mechanisms (Chang and Tsai, 2002; Kirchner, 2000; Mathews, 1999; Shyu, 1998) are the main causes of policy intent distortion.	(Brueck et al., 2025; Chang et al., 2021; Chang and Tsai, 2002; Chen and Wang, 2023; Chiang, 2000; Choi, 2014; Fauth et al., 2024; Huang and Malkin, 2025; Khan, 2024; Kirchner, 2000; Konstari and Valkokari, 2025; Lee and von Tunzelmann, 2005; Mathews, 1999; Meng et al., 2025; Shyu, 1998; Song and Wen, 2023; Spencer and Grindley, 1993; Stokols and Kollar, 2025; Tong and Wan, 2023; Wang et al., 2024; Zhang et al., 2023)
Micro	(RQ3) What are the endowments of the regional context (RIS)?	Static endowments (assets/institutions) Dynamic capabilities (absorptive/coupling/resilience)	Policy effectiveness is highly “context-dependent.” Regional endowments provide “path dependency” constraints (Huggins et al., 2023; Johnston and Huggins, 2023; Lee and von Tunzelmann, 2005; Yang and Yun, 2023), while regional dynamic capabilities (Dawley, 2007; Kirchner, 2000; Lin and Rasiah, 2014; Mathews, 1999) are the key to achieving “path unlocking” (Bhattacharya et al., 2017; Rolf et al., 2025).	(Baark and Qian, 2025; Bhattacharya et al., 2017; Chang et al., 2021; Chang and Tsai, 2002; Choi, 2014; Dawley, 2007; Fauth et al., 2024; Hsu, 2004; Huggins et al., 2023; Johnston and Huggins, 2023; Khan, 2024; Kirchner, 2000; Lee, 2003; Lee and von Tunzelmann, 2005; Lin and Rasiah, 2014; Mathews, 1999; Rasiah et al., 2016b; Rolf et al., 2025; Shiu et al., 2014; Shyu, 1998; Spencer and Grindley, 1993; Stokols and Kollar, 2025; Tong and Wan, 2023; Wang et al., 2024; Yang and Yun, 2023)

#### 4.2.1 Fit 1: “strategy-execution fit” (macro-meso alignment)

The first critical fit occurs between the macro-level national strategic intent (structure) and the meso-level policy implementation mechanisms (process). It demands that a state's chosen “Governance” model and “Policy Tools” (Meso) must be aligned with its pursued “Strategic Goal” (Macro). If the meso-level implementation system cannot support the macro-level strategic ambition, the first step of policy from intention to action is likely to fail. The evidence in the literature amply demonstrates the consequences of such a “Misfit.” For example, the current macro-strategy of many nations has shifted to being “security-driven” (Hsu, 2017; Park, 2023), an objective (e.g., supply chain “autonomy and controllability”) that often conflicts with traditional “market efficiency” logic. This internal tension places extremely high demands on meso-level governance (RQ2). In the case of China's Mainland, a significant “Misfit” emerged between the macro-level catch-up strategy (RQ1) and the meso-level central-local governance mechanisms (RQ2). The central government's macro-planning (like the “Big Fund”) was intended for “path creation” (Huang and Malkin, 2025; Khan, 2024; Meng et al., 2025), but it was distorted during implementation by the meso-level “tournament-style” competition among local governments (Meng et al., 2025; Song and Wen, 2023; Tong and Wan, 2023). The local governments' incentive mechanisms (Meso Governance) were not fully aligned with the national strategy's goal of “technological breakthrough” but were instead more inclined toward “scrambling for projects” and “expanding investment,” ultimately leading to severe resource misallocation and overbuilding risks (Meng et al., 2025). This is a typical “Strategy-Execution Misfit.” Conversely, the successful cases in the literature demonstrate a high degree of “Strategy-Execution Fit.” The success of the “Developmental State” strategy (RQ1) in Taiwan, Province of China (Hwang and Choung, 2014) was keyed on its selection of a perfectly adapted meso-level governance mechanism (RQ2): the semi-public “peripheral agency” ITRI, which was institutionally “detached” from the conventional bureaucracy (Billing et al., 2024). This governance arrangement (Meso) proved to be the ideal vehicle for executing the high-risk strategy of technological incubation (Macro) (Billing et al., 2024; Chang et al., 2021; Chang and Tsai, 2002). Likewise, Singapore's success is also attributed to the high alignment between its macro-strategy (embedding in GVCs) and its efficient, professional meso-level governance agency (the EDB) (Mathews, 1999).

#### 4.2.2 Fit 2: “execution-context fit” (meso-micro alignment)

The second critical fit occurs between the top-down policy intervention (process) and the policy's landing point (contextual structure). It demands that the meso-level policy instruments and governance mechanisms (RQ2) must match the specific context (RQ3) of the RIS being intervened in. This fit is at the core of the “place-based” policy philosophy (Baumgartinger-Seiringer et al., 2025; Rolf et al., 2025). Policy instruments are not universal; they must be “embedded” within the region's specific endowments and capabilities. As related regional theory emphasizes, policy paths, such as for emissions reduction, necessarily differ across regions due to heterogeneity (Bhattacharya et al., 2017), and the same holds true for semiconductor industrial policy. Most policy failures in the literature can be attributed to this “Execution-Context Misfit.” The failure of the “flagship project” in North East England (Kirchner, 2000) is the most profound lesson. A meso-level policy tool (Meso Tool, i.e., introducing the Siemens fab) was “air-dropped” into a micro-context (Micro Context), an old industrial area lacking the requisite industrial and institutional endowments (RQ3). When the meso-intervention (RQ2) cannot couple with the micro-level “static endowments” and “dynamic capabilities,” policy outcomes are highly likely to be compromised. Comparative research on the development of different regions in Taiwan, Province of China, reached a similar conclusion: the reason meso-level interventions (like science parks) were effective in some areas but not others was that the key explanatory variable was whether the micro-region (RQ3) possessed a sufficient stock of human capital and local firm R&D investment (i.e., absorptive capacity) (Rasiah et al., 2016a). In contrast, successful regional revivals invariably demonstrate the wisdom of “Execution-Context Fit.” The revival of Germany's “Silicon Saxony” (Johnston and Huggins, 2023) was not creatio ex nihilo. Rather, the meso-level policy tools (RQ2, e.g., R&D networks and cluster support) achieved a perfect fit with the micro-electronics “industrial heritage” and skilled worker base (Micro Endowment, RQ3) left over from the GDR period (Yang and Yun, 2023). Similarly, the MINATEC center in Grenoble, France (Meso Tool, RQ2) was also established around the region's unique endowment (Micro Asset, RQ3) of world-class micro- and nanotechnology research institutions (Johnston and Huggins, 2023). The early comparison of South Korea and Saxony, Germany, also found that the two adopted starkly different meso-support paths (RQ2), precisely to adapt to their heterogeneous micro-contexts (RQ3)—one “dominated by large enterprises” and the other “dominated by SME networks” (Choi, 2014; Yang and Yun, 2023). In summary, policy effectiveness is a systematic undertaking of “Dual Fit,” requiring policymakers to succeed on both dimensions simultaneously.

### 4.3 Cross-case validation: a process tracing analysis of mechanism fit

To validate the explanatory power of the proposed “dual fit” framework, we employ a “process tracing” approach (Bennett and Checkel, 2015) to analyze three representative cases identified in the systematic review. Unlike statistical correlation, process tracing allows us to open the black box of causality by identifying the specific diagnostic evidence that links macro-structures to micro-outcomes. By tracing the causal mechanisms within these distinct contexts, we verify how different configurations of “structure” (strategy/endowments) and “process” (governance) lead to divergent “functions” (resilience/outcomes).

#### 4.3.1 Case A: high dual fit—the ITRI model in Taiwan, Province of China

The development of the semiconductor industry in Taiwan, Province of China, represents a paradigmatic case of “high dual fit,” where meso-level governance effectively bridged the gap between macro-strategy and micro-endowments.

Structural Alignment: At the macro-level, the region adopted a “developmental state” strategy aimed at technological catch-up. However, unlike the South Korean model which relied on large conglomerates, the micro-level endowment of Taiwan, Province of China was characterized by a fragmented network of SMEs with high flexibility but limited individual R&D capacity (Lin and Rasiah, 2014; Shiu et al., 2014).

Process Mechanism: To mediate this structure, the governance process relied on the ITRI. As a “peripheral agency” institutionally detached from rigid bureaucracy (Hsu, 2004), ITRI functioned as a critical ecosystem orchestrator. It did not merely distribute subsidies; instead, it centralized the absorption of advanced foreign technologies (such as RCA's CMOS process) and then diffused them to the private sector through spin-offs like UMC and TSMC (Chang et al., 2021; Chang and Tsai, 2002).

Functional Outcome: This governance mechanism achieved a perfect “execution-context fit” by aligning with the region's human capital structure. The result was a robust “Adaptive Function,” enabling the region to evolve from a latecomer to a global manufacturing hub with a resilient, cluster-based innovation system (Wong and Lee, 2022).

#### 4.3.2 Case B: governance coordination challenge—the “big fund” model in China's Mainland

The case of China's Mainland illustrates the complexities and coordination challenges inherent in a rapid, state-led catch-up strategy under a multi-level governance structure.

Structural context: In response to geopolitical pressures, the national strategy shifted decisively toward “technological sovereignty” and “supply chain resilience” (RQ1). This macro-intent was operationalized through massive state capital mobilization, most notably the National Integrated Circuit Industry Investment Fund (the “Big Fund”) (Meng et al., 2025; Tong and Wan, 2023).

Process dynamics: While the resource injection was substantial, the literature identifies a tension in the “meso-process” of implementation. The governance structure relies heavily on a hierarchy where central strategic goals are executed by local governments. This structure often triggers a “tournament-style” competition (Song and Wen, 2023), where local authorities compete aggressively for semiconductor projects to drive GDP growth.

Mechanism analysis: Diagnostic evidence suggests a partial misalignment in “strategy-execution fit.” The incentive mechanism for local governments sometimes prioritized short-term scale expansion over long-term technological breakthrough, leading to risks of redundant construction in regions that lacked the necessary industrial foundations (Khan, 2024; Meng et al., 2025). However, recent policy adjustments emphasize the construction of “state-business-university” tripartite networks (Chang et al., 2025), indicating an adaptive evolution toward better coordination and higher investment efficiency.

#### 4.3.3 Case C: contextual misfit—the “flagship project” in North East England

The experience of the North East England region (specifically the Siemens project) provides a cautionary tale of “contextual misfit,” demonstrating that policy instruments cannot succeed without local embedding.

Governance process: The primary meso-level policy instrument employed was the attraction of Foreign Direct Investment (FDI) through financial incentives. The local governance bodies successfully attracted a global “Flagship Project”—a Siemens wafer fabrication plant—aiming to jumpstart regional re-industrialization (Kirchner, 2000).

Contextual structure: However, the micro-region was an old industrial area that lacked the requisite “institutional thickness” and specialized supplier networks specific to the semiconductor industry (Dawley, 2007).

Functional outcome: Process tracing reveals that due to this lack of local embeddedness, the region failed to absorb knowledge spillovers from the flagship plant. The semiconductor activities remained an “enclave” rather than an ecosystem. Consequently, when the external firm faced market shifts and divested, the region failed to retain the industrial capabilities, leading to a collapse of the initiative. This confirms that without “Execution-Context Fit,” meso-level tools (even powerful ones like FDI attraction) may fail to generate sustainable resilience functions.

#### 4.3.4 Summary of cross-case diagnosis

To synthesize these findings, Table 7 presents a diagnostic comparison of the three cases based on the SPF framework. It highlights how the alignment (or misalignment) of governance mechanisms with structural contexts determines the final policy outcomes.

**Table 7:** Cross-case diagnostic analysis of policy-ecosystem fit.

**Case context**	**Macro-structure (strategy)**	**Meso-process (governance)**	**Micro-context (endowments)**	**Fit configuration**	**Functional outcome**
Taiwan, Province of China (ITRI case)	Developmental/catch-up	Mediated network: semi-public agency (ITRI) bridging state and market.	High absorptive capacity: dense SME networks and skilled labor.	High dual fit (aligned governance and context)	Adaptive resilience: sustainable upgrading and cluster formation.
China's Mainland (Big Fund case)	Sovereignty/path creation	Multi-level hierarchy: state capital injection + local competition.	Heterogeneous: varied endowments across regions; coordination complexity.	Coordination challenge (macro-meso tension)	Capacity expansion: rapid scale-up, requiring enhanced coordination efficiency.
UK North East (Siemens case)	FDI attraction/regional dev.	Exogenous injection: subsidies for flagship FDI projects.	Low embeddedness: weak local supply chain and institutional thickness.	Contextual misfit (meso-micro disconnect)	Transient growth: vulnerability to external divestment shocks.

### 4.4 Theoretical contributions and policy implications

#### 4.4.1 Theoretical contributions: constructing a middle-range theory of governance

This study makes a distinct theoretical contribution to the field of engineering management and public policy by filling the “Meso-level Gap” identified in the introduction through the construction of a Mertonian Middle-Range Theory (Abner et al., 2017) of semiconductor governance. Existing literature has long bifurcated between macro-geopolitical narratives and micro-corporate strategies, leaving the transmission mechanism of policy largely unexplained. Our framework integrates these levels by adopting the SPF perspective (Liu and Fan, 2017). First, we conceptualize national strategy not merely as political intent but as a static “Institutional Structure” that defines the legitimacy and boundaries of intervention. Second, we reframe meso-level governance not as a passive toolbox but as a dynamic “Process” that must mediate between the rigidity of macro-structures and the heterogeneity of micro-contexts. This shifts the analytical focus from a static inventory of policy tools to the dynamic alignment of governance mechanisms, providing a more granular explanation for why similar macro-strategies yield divergent industrial outcomes across different regions.

Furthermore, this study moves beyond descriptive categorization to provide a mechanistic explanation of policy effectiveness. By distinguishing the specific causal pathways of “Strategy-Execution Fit” and “Execution-Context Fit,” the “Dual Fit” model clarifies the internal logic of policy implementation failure. It reveals that “Governance Failure” is fundamentally a misalignment between the incentive structures of the macro-strategy and the meso-process, while “Contextual Failure” is a disconnect between the top-down governance process and the region's bottom-up absorptive capacity. This mechanistic view extends recent engineering management scholarship on complex systems (Zhang et al., 2025), offering a diagnostic tool for analyzing the resilience of strategic industries in a fragmented global order.

#### 4.4.2 Implications for policymakers and engineering managers

The “dual fit” framework offers critical practical implications for stakeholders navigating the current “security-driven” era. For policymakers, the findings suggest a fundamental shift from “target-setting” to “process-governance.” The prevalence of “contextual misfit” in our review—exemplified by the case of North East England—serves as a stark warning against “one-size-fits-all” policy isomorphism. Policymakers should consider abandoning the impulse to blindly replicate successful models like the Hsinchu Science Park without a realistic audit of local “static endowments” and “dynamic capabilities.” Instead, interventions must be “place-based” (Baumgartinger-Seiringer et al., 2025), tailored to the specific institutional thickness of the region. Moreover, the challenge of “Macro-Meso Fit” implies that the central government's primary task should extend beyond funding to designing governance mechanisms that can effectively constrain local opportunism and align regional incentives with national security goals (Meng et al., 2025).

For engineering managers, the framework highlights the necessity of developing “adaptive governance” capabilities. In an environment characterized by geopolitical friction, firm strategy can no longer be decoupled from state logic. Managers should evaluate potential investment locations not merely by the generosity of financial subsidies (a Meso variable) but by the region's “Execution-Context Fit”—specifically, its institutional thickness and workforce absorptive capacity (Micro variables). This diagnostic approach enables firms to identify and avoid “policy traps”—regions with high subsidy promises but low ecosystem resilience—and instead prioritize locations where meso-level governance effectively orchestrates micro-level capabilities, thereby ensuring long-term operational resilience.

## 5 Conclusion and future research prospects

This study's departure point stemmed from a core puzzle: In the new wave of global semiconductor industry policy, why do similar macro-intentions produce starkly divergent industrial outcomes in different regions? To answer this question, this study employed the methods of Systematic Review and Narrative Synthesis to conduct a multi-level analysis of 104 core articles from the WoS Core Collection. This paper's central argument is that the effectiveness of regional semiconductor policy is a problem of “Multi-level Fit.” Drawing on the SPF perspective (Liu and Fan, 2017), we propose a Mertonian Middle-Range Theory (Abner et al., 2017) termed the “dual fit” framework. We argue that policy success depends not on the “optimal” design of any single dimension, but on the simultaneous achievement of “strategy-execution fit” (aligning macro-structure with meso-process) and “execution-context fit” (embedding meso-process into micro-context). By integrating national strategy, governance mechanisms, and regional endowments into this unified mechanistic model, this paper provides a new mechanistic explanation for understanding the heterogeneous outcomes of current global semiconductor policy interventions.

This study's main theoretical contribution lies in bridging the “meso-level theoretical gap” in the existing literature between the macro-level (state competition) and the micro-level (corporate strategy), using the “meso-governance process” as a critical perspective. This framework moves beyond a simple “inventory” of policy tools; it reveals two core mechanisms of policy failure: “governance failure” (i.e., a macro-meso mismatch, such as central-local coordination failure) and “contextual failure” (i.e., a meso-micro mismatch, such as a disconnect between policy and regional absorptive capacity). This finding holds significant practical implications for global policymakers and engineering managers currently operating under the “security-driven” macro-paradigm: the core challenge of policy design has shifted from “target setting” to the “governance of fit.” Consequently, any “one-size-fits-all” intervention that ignores regional context (endowments and capabilities) faces significant implementation risks.

As a systematic review based on extant literature, this study's limitation is that its analytical depth is constrained by the existing focus of the sampled articles. Although we have systematically synthesized the importance of “fit,” ample space remains for future empirical research. First, this study calls for deepening the “process tracing” approach through longitudinal fieldwork. While this study provided a preliminary diagnostic analysis, we still know little about the micro-politics of how governance mechanisms are negotiated inside the “black box” of institutions. Future studies should employ in-depth interviews to reveal how actors navigate the tensions of the new “security-driven” paradigm (e.g., in “friend-shoring” initiatives).

Second, future research should seek deeper insights from “comparison” and methodological diversification. This study has categorized different regional “meso-micro fit” models (e.g., Saxony, Germany, and Hsinchu, Taiwan, Province of China), but the future requires more refined “paired comparison” studies to examine how similar “meso” interventions produce differentiated evolutionary paths in different “micro” contexts. Moreover, scholars should move beyond qualitative synthesis to employ quantitative techniques, such as fuzzy-set Qualitative Comparative Analysis (fsQCA) (Chen et al., 2024), to rigorously test which configurations of governance and endowment yield the highest resilience.

Finally, the “security-driven” macro-shift is a historical process still in motion. Its long-term structural impacts on global value chains and regional innovation ecosystems remain to be seen (Manning and Richter, 2023). Future inquiries must therefore expand the analytical horizon to include emerging paradigms like Industry 6.0, exploring how AI-embedded modalities might further reshape the strategic road-mapping and governance of public interest technologies (Carayannis et al., 2024). This represents the most urgent and challenging agenda for future engineering management research.

## Data Availability

Publicly available datasets were analyzed in this study. This data can be found here: https://doi.org/10.5281/zenodo.18447066.
