# Supplementary material for: Managing technological sovereignty: a systematic review of semiconductor industry policy and regional ecosystem governance
Source: Front Res Metr Anal. 2026 Mar 6;11:1762083. doi: 10.3389/frma.2026.1762083 (PMC13002567; doi:10.3389/frma.2026.1762083)
Supplement: Supplementary file 1 [file Data_Sheet_1.docx]

**Appendix A: Full Search Strategy (Web of Science Core Collection)**

**Database:** Web of Science (WoS) Core Collection

**Date of Search:** October 26, 2025

**Search Field:** Topic (TS) = Title, Abstract, Author Keywords, Keywords Plus

**Language:** English

**Document Types:** Article; Proceedings Paper

**Search 1: Core Intersection (Industry + Policy + Region)**

TS=(semiconductor* OR "integrated circuit*" OR chip* OR microelectronic* OR ICs OR foundry OR fabless OR "wafer fab*") AND TS=(policy OR policies OR "industrial policy" OR "public policy" OR governance OR "policy instrument*" OR subsidy* OR incentive* OR "export control*" OR "multi-level governance" OR MLG OR "policy network*") AND TS=(region* OR "local" OR cluster* OR "geograph*" OR agglomeration* OR spatial OR "place-based" OR "science park*" OR "technology park*" OR "innovation system*" OR "regional innovation system*" OR RIS OR ecosystem* OR "absorptive capacity" OR "path dependenc*")

**Search 2: Macro-Strategic Supplement**

TS=(semiconductor* OR "integrated circuit*" OR chip* OR microelectronic* OR ICs OR foundry OR fabless OR "wafer fab*") AND TS=("developmental state" OR "entrepreneurial state" OR "state role*" OR "state strategy" OR "national strategy" OR "state intervention*" OR "state-led" OR "national security" OR "geopolitic*" OR "techno-nationalism" OR "supply chain resilience") AND TS=(region* OR "local" OR cluster* OR "geograph*" OR agglomeration* OR spatial OR "place-based" OR "science park*" OR "technology park*" OR "innovation system*" OR "regional innovation system*" OR RIS OR ecosystem*)

**Search 3: National Policy Supplement (Five Sub-themes)**

Logic: Identifies national-level policy studies. To ensure coverage of diverse governance dimensions, this search was executed via five distinct sub-queries based on the keyword categories present in the policy string.

Base Industry String (Applied to all sub-queries):

TS=(semiconductor* OR "integrated circuit*" OR chip* OR microelectronic* OR ICs OR foundry OR fabless OR "wafer fab*")

Sub-query 3.1 (General Policy):

AND TS=(policy OR "industrial policy" OR "public policy")

Sub-query 3.2 (Policy Instruments):

AND TS=("policy instrument*" OR subsidy* OR incentive*)

Sub-query 3.3 (Governance & Networks):

AND TS=(governance OR "multi-level governance" OR "policy network*")

Sub-query 3.4 (Geopolitics & Control):

AND TS=("export control*" OR "techno-nationalism" OR "geopolitic*")

Sub-query 3.5 (State Theory):

AND TS=("developmental state" OR "entrepreneurial state" OR "state strategy")

**Search 4: Theoretical Supplement (Innovation Mechanisms)**

TS=(semiconductor* OR "integrated circuit*" OR chip* OR microelectronic* OR ICs OR foundry OR fabless OR "wafer fab*") AND TS=("innovation system*" OR "regional innovation system*" OR RIS OR "innovation ecosystem*" OR "knowledge spillover*" OR "absorptive capacity" OR "path dependenc*" OR "institutional thickness")
